# Neural Loop by the Median Nerve’s Roots Associated with Multiple Neurovascular Anomalies: A Cadaver-based Case Report with Clinical Aspects

**DOI:** 10.7759/cureus.6163

**Published:** 2019-11-15

**Authors:** George K Paraskevas, Konstantinos N Koutsouflianiotis, Irene Asouhidou, Kalliopi Iliou, George Noussios

**Affiliations:** 1 Orthopaedics, Aristotle University of Thessaloniki, Thessaloniki, GRC; 2 Internal Medicine, General Hospital of Thessaloniki "G. Gennimatas", Thessaloniki, GRC; 3 Anatomy, Aristotle University of Thessaloniki, Thessaloniki, GRC; 4 Psychiatry, Aristotle University of Thessaloniki, Thessaloniki, GRC; 5 Physical Education and Sports Sciences, Aristotle University of Thessaloniki, Thessaloniki, GRC

**Keywords:** neural loop, median nerve, superficial brachial artery, musculocutaneous nerve’s anastomosis

## Abstract

The existence of a combination of neural and vascular variations in the axilla and arm region are relatively common. In the current case study, an association of a neural loop by the roots of the left median nerve along with an ipsilateral proximal division of the brachial artery in the upper arm and bilateral communications between the median and musculocutaneous nerves is documented. The morphological features of these abnormalities, along with the clinical implications induced during nerve blocks and surgical interventions in the region, are discussed as well.

## Introduction

The presence of neurovascular anomalies of the axilla and arm region is not so rarely detected. During the embryological development of the upper extremity, the growing nerves and vessels are intimately related. It has been postulated that the aberrant arteries of the aforementioned regions induce the development of abnormalities in the components parts of the brachial plexus [1]. Neural loops are uncommon variants of nerves. Various neural loops concerning the formation of the median nerve (MN) have been documented without content or penetrated by arteries such as the superficial brachial artery [2]. The abnormal proximal division of the brachial artery gives rise to a so-called superficial brachial artery, an arterial variation that has an incident rate of 22% [3]. Ultimately, the existence of an anastomosis between the MN and musculocutaneous nerve (aMN - MCN) can be detected with a frequency from 5% to 53.6% [4-5].

In the current study, we display a very rare combination of neurovascular anomalies, consisting of a neural loop at the site of the MN’s formation, an aberrant superficial brachial artery along with bilateral anastomosis between MN and MCN (aMN - MCN), discuss their anatomical features, provide a short review of the relevant literature, and deal with the clinical implications.

## Case presentation

**Figure 1:**
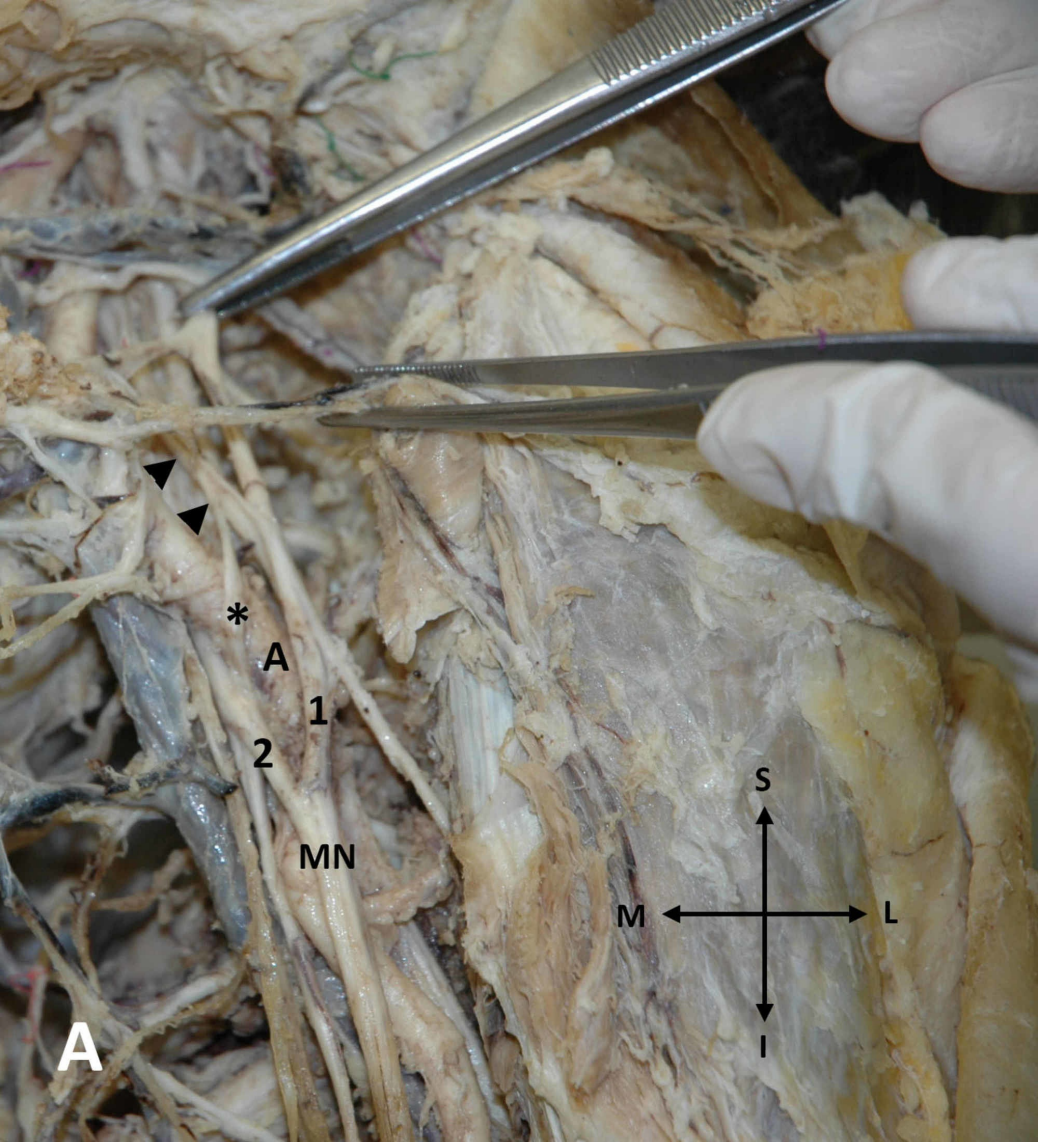
Additional lateral MN's root on the left arm An additional lateral root (asterisk) of the left median nerve (MN) originating from the anterior division of the middle trunk of the brachial plexus (arrowheads) and fusing with the medial root (2) of the left MN (1: lateral root of the MN, A: axillary artery)

During our routine dissection studies and after utilizing a conventional dissecting set in an embalmed 72-year-old male cadaver, multiple bilateral arterial and nervous variants in the axilla and arm region were encountered. The cause of the cadaver’s death was unrelated to the current study. In particular, after meticulous dissection of the axilla and arm region, bilaterally, we came across in the left axilla, an additional lateral root of the MN, arising from the terminal portion of the anterior division of the middle trunk of the brachial plexus and fusing with the medial root of the left MN, thus forming a neural loop. That additional lateral MN’s root was coursing the anterior aspect of the third portion of the left axillary artery; the formed neural loop was not penetrated by some anatomical structure (Figure 1A).

**Figure 2:**
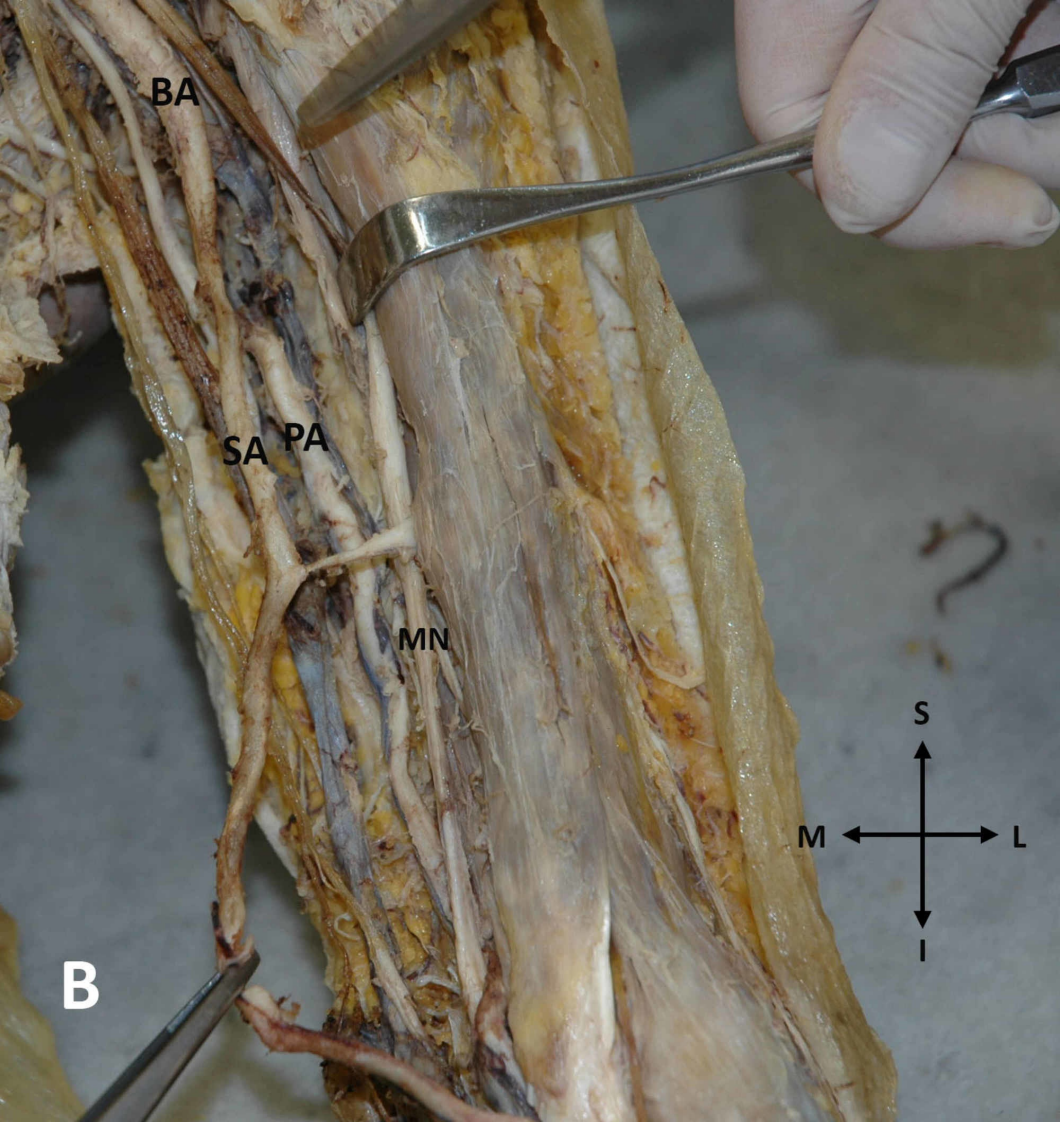
Proximal division of the brachial artery on the left arm Early division of the left brachial artery (BA) into a superficial brachial artery (SA) and a profunda brachial artery (PA) (MN: median nerve) (S: superior, I: inferior, L: lateral, M: medial)

 Moreover, on the left arm, a proximal division of the brachial artery in the upper third of the arm was encountered (Figure 2).

**Figure 3:**
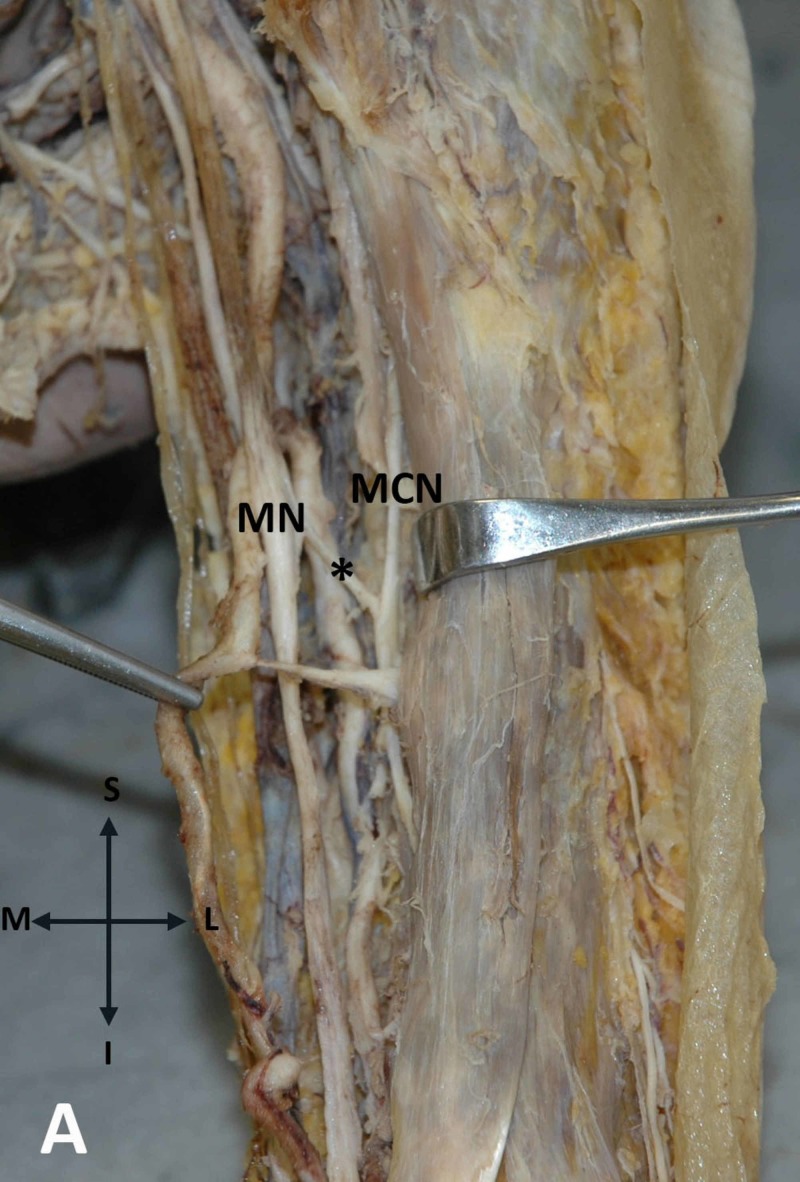
Anastomosis between the MN and MCN on the left upper arm The communicating branch (asterisk) between the median nerve (MN) and the musculocutaneous nerve (MCN) in the left upper arm is demonstrated

The brachial artery divided into a superficial brachial artery continuing distally as the radial artery and the profunda brachial artery that continued as the ulnar artery in the forearm. Furthermore, an anastomosis between MN and MCN (aMN - MCN) bilaterally was detected. Particularly, on the left upper arm, such as anastomosis was observed originating from the MN and directed obliquely laterally in order to merge with the ipsilateral MCN (Figure 3).

**Figure 4:**
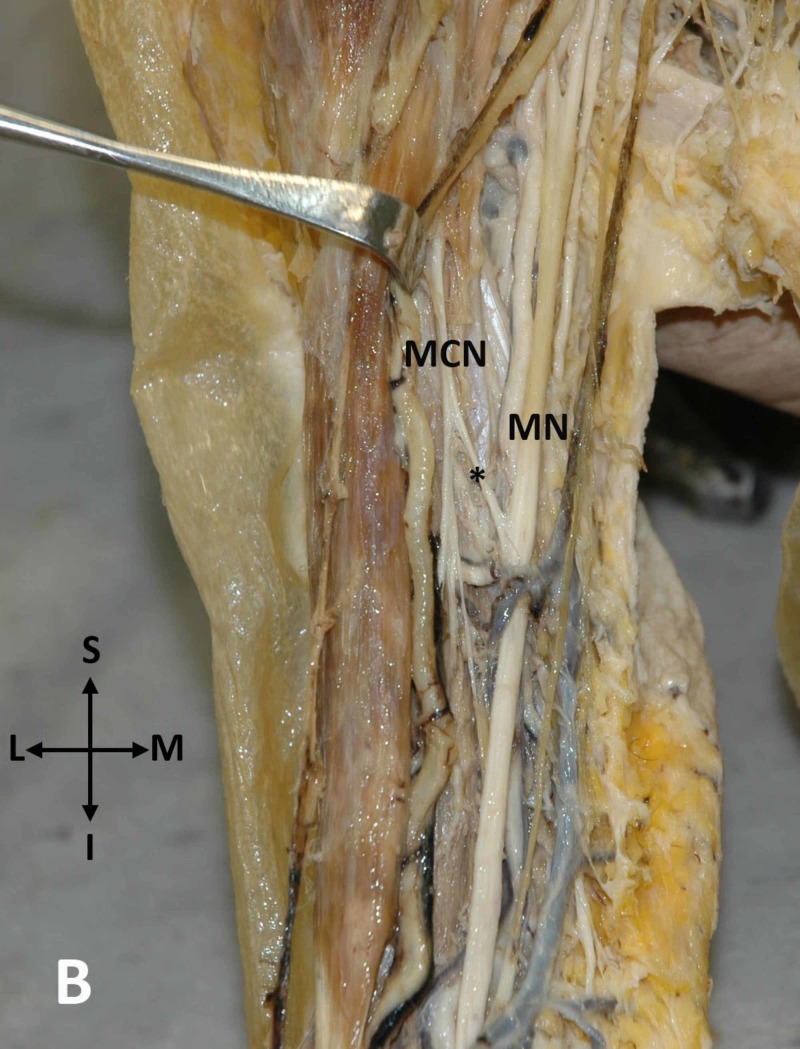
An anastomosis between the MCN and MN on the right upper arm An anastomosis (asterisk) between the musculocutaneous nerve (MCN) and the median nerve (MN) in the right upper arm is seen (S: superior, I: inferior, L: lateral, M: medial)

On the contrary, on the right upper arm, the aMN - MCN was directed from the MCN medially to fuse with the trunk of the ipsilateral MN (Figure 4).

No other anatomical abnormalities, traumatic injuries, or evidence of previous surgical procedures were documented on the axilla and arm region bilaterally. The morphology and topography of the observed neurovascular anomalies were recorded by a digital camera, Nikon D3400 (Nikon Corporation, Tokyo, Japan).

## Discussion

Variable loops of neural trunks have been described, sometimes being pierced by other anatomical structures, usually vessels. Among these nerves is the radial nerve penetrated by an aberrant axillary artery [6] or subscapular artery [7], the MN penetrated by a muscular branch of the brachial artery in the distal half of the arm [8] or a variant palmaris profundus muscle in the lower portion of the forearm [9], the ulnar nerve penetrated at the carpal region and Guyon canal by the trunk of the ulnar artery [10], and the palmar digital nerves penetrated by their common digital arteries [11]. Such neural loops can be asymptomatic. However, in cases where neural loops are penetrated by arteries, the progressive pulsatile arterial flow may induce static compression of the neural loop, leading to motor or land sensory symptoms.

The typical MN’s formation by the union of its medial and lateral roots occurs in approximately 85% of specimens [12]. MN can be formed by the fusion of three roots, usually, two originating from the lateral cord of the brachial plexus and one from the medial cord [13], four roots [14], or even five roots [15]. In the current study, an additional root arose from the anterior division of the middle primary trunk of the brachial plexus and after coursing anterior to the third part of the axillary artery, it was fused with the medial root of the MN. Studies suggest that aberrant branches that cross the axillary artery may induce compression, resulting in symptoms of ischemia. [16]. It has also been claimed that such supernumerary communications between the lateral and medial cords are susceptible to lesions during surgical interventions in the axilla and upper arm [17].

Furthermore, in our study, that abnormal MN’s formation was accompanied by a superficial brachial artery arising from the axillary artery at the proximal third of the arm coursing superficial to the MN. That pattern where the brachial artery divides in the arm and sends one arterial stem anterior to the MN and the other one posterior to the MN occurs in 8% of the population [3]. Moreover, studies suggest that the relationship between the brachial plexus and axillary artery departs from the normal configuration in 8% of observations. It has been documented that abnormalities in the component parts of the brachial plexus are most frequently caused by aberrant arteries [1]. The incidence of the superficial brachial artery is estimated to be as high as 22% [3].

In our current research, we observed the presence of a bilateral aMN - MCN. The incidence of such an anastomosis varies between 5% [4] and 53.6% [5]. The bilateral occurrence of aMN - MCN, as noticed in our case report, was observed in 7.6% of 79 cadavers [18]. Most usually, aMN - MCN is directed from the MCN distally and medially to MN, whereas, very rarely, it is directed from the MN to MCN as it is noted in the left arm of our case report [12]. In the latter case, MCN receives from MN nerve fibers for the biceps brachii, the brachialis muscle, and the skin of the lateral aspect of the forearm. Thus, in the case of MCN damage proximal to aMCN - MN, motor and sensory disturbances should be potentially limited. On the contrary, when an MN lesion occurs proximal to the aMCN - MN in case of an aMCN - MN that is directed from the MCN to MN, the MN’s motor and sensory deficits distally may be restricted.

## Conclusions

The awareness of neural and arterial variants is crucial for surgeons and anesthesiologists who perform procedures in the axilla, in order to avoid damage to these structures. The neural loop and communication between the MN and MCN could further complicate motor and sensory deficits following surgical trauma or damage to these structures.

## References

[1] Miller RA (1939). Observations upon the arrangement of the axillary artery and brachial plexus. Am J Anat.

[2] Paraskevas G, Varnalidis I, Koutsouflianiotis K (2017). Median nerve’s loop in the arm penetrated by a superficial brachial artery: case report and neurosurgical considerations. Int J Res Med Sci.

[3] Lippert H, Pabst R (1985). Arterial Variations in Man. Classification and Frequency.

[4] Beheiry EE (2004). Anatomical variations of the median nerve distribution and communication in the arm. Folia Morphol.

[5] Guerri-Guttenberg RA, Ingolotti M (2009). Classifying musculocutaneous nerve variations. Clin Anat.

[6] Honma S, Kawai K, Koizumi M, Yoshinaga K, Tanii I, Kodama K (2004). An aberrant axillary artery penetrating the origin of the radial nerve from deep to superficial. Ann Anat.

[7] Kuwar Kuwar, R R, Basnet K, Dhungel S, Thapa T (2010). Penetration of radial nerve by subscapular artery. JIOM.

[8] Roy TS (2003). Median nerve penetration by a muscular branch of the brachial artery. Clin Anat.

[9] Chou HC, Jeng H, Ko TL, Pai MH, Chang CY, Wu CH (2001). Variant palmaris profundus enclosed by an unusual loop of the median nerve. J Anat.

[10] Olave E, Del Sol M, Gabrielli C, Prates JC, Rodrigues CF (1997). The ulnar tunnel: a rare disposition of its contents. J Anat.

[11] Lee JY, Kim YR, Kim JN, Choi HG, Song WC, Koh KC (2010). Penetration of the digital nerves by the common palmar digital arteries in human cadavers. J Hand Surg.

[12] Tubbs RS, Shoja MM, Loukas M (2016). Bergman’s Comprehensive Encyclopedia of Human Anatomic Variation. https://books.google.co.in/books?hl=en&lr=&id=6h1EDQAAQBAJ&oi=fnd&pg=PR11&dq=Bergman%E2%80%99s+comprensive+encyclopedia+of+human+anatomic+variation&ots=HGRvrm6njQ&sig=Kmieuh9VywzOxAp5P3pGuT_sZiY&redir_esc=y#v=onepage&q=Bergman%E2%80%99s%20comprensive%20encyclopedia%20of%20human%20anatomic%20variation&f=false.

[13] Paraskevas G, Lazaridis N, Piagkou M, Natsis K (2018). Superficial brachial artery traversing a median nerve loop in the arm associated with other vascular and muscular anomalies: case report and clinical implications. Ital J Anat Embryol.

[14] Uzun A, Seelig LL Jr (2001). A variation in the formation of median nerve and communicating branches between musculocutaneous and median nerve. Folia Morphol (Warsz).

[15] Natsis K, Paraskevas G, Tzika M (2016). Five roots pattern of median nerve formation. Acta Med.

[16] Das S, Paul S (2005). Anomalous branching pattern of lateral cord of brachial plexus. Int J Morphol.

[17] Uzun A, Bilgic S (1999). Variations in the formation of brachial plexus in infants. Turk J Med Sci.

[18] Venieratos D, Anagostopoulou S (1998). Classification of communications between the musculocutaneous and median nerves. Clin Anat.

